# TCF12 Activates TGFB2 Expression to Promote the Malignant Progression of Melanoma

**DOI:** 10.3390/cancers15184505

**Published:** 2023-09-11

**Authors:** Youjia Tian, Jiang Zhou, Xinxin Chai, Zejun Ping, Yurong Zhao, Xin Xu, Chi Luo, Jinghao Sheng

**Affiliations:** 1 Affiliated Hangzhou First People’s Hospital, Zhejiang University School of Medicine, Hangzhou 310006, China; tianyj@zju.edu.cn (Y.T.); 12018090@zju.edu.cn (X.C.); 22118833@zju.edu.cn (Z.P.); 0620803@zju.edu.cn (Y.Z.); xxuxin@zju.edu.cn (X.X.); 2 Liangzhu Laboratory, Zhejiang University, Hangzhou 310012, China; 3 Cancer Center, Zhejiang University, Hangzhou 310058, China; 22018055@zju.edu.cn; 4 Zhejiang Provincial Key Laboratory of Bioelectromagnetics, Zhejiang University School of Medicine, Hangzhou 310058, China

**Keywords:** melanoma, TCF12, RNA-seq, TGFB2, BRAF(V600E)

## Abstract

**Simple Summary:**

Melanoma is the deadliest form of skin cancer, with the BRAF(V600E) mutation being the most prevalent driver mutation. Despite targeted therapies against BRAF(V600E) mutation and immune checkpoint-blocking antibodies providing treatment options for patients, the heterogeneous nature of melanoma significantly limits treatment efficacy. Understanding diverse regulatory mechanisms in melanoma will shed light on improving the current treatment modalities. In this study, we explored the function of a novel transcriptional activator, TCF12, in melanoma progression. We found that the expression level of TCF12 is elevated as melanoma progresses, and high expression is strongly associated with poor survival outcomes in melanoma patients. Functionally, TCF12 enhances melanoma proliferation and metastasis, as well as the sensitivity to BRAF(V600E)-targeted therapy. Mechanistically, TGFB2 is the direct transcriptional target of TCF12, mediating its pro-tumorigenic effects. Collectively, our study supported the oncogenic functions of TCF12 in melanoma, revealing it as a potential target to improve the efficacy of BRAF(V600E)-targeted therapy.

**Abstract:**

As one of the most common malignant tumors, melanoma is a serious threat to human health. More than half of melanoma patients have a BRAF mutation, and 90% of them have a BRAF(V600E) mutation. There is a targeted therapy for patients using a BRAF(V600E) inhibitor. However, no response to treatment is generally inevitable due to the heterogeneity of melanoma. Coupled with its high metastatic character, melanoma ultimately leads to poor overall survival. This study aimed to explore the possible mechanisms of melanoma metastasis and identify a more effective method for the treatment of melanoma. In this paper, we report that TCF12 expression is higher in melanoma, especially in metastatic tumors, through analyzing data from TCGA. Then, cell proliferation, colony formation, and transwell assays show that the upregulated expression of TCF12 can promote proliferation and metastasis of melanoma cells in vitro. The same result is confirmed in the subcutaneous tumor formation assay. Moreover, TGFB2 is identified as a direct downstream target of TCF12 by RNA-seq, qPCR, immunoblotting, ChIP, and a dual luciferase reporting assay. Interestingly, depletion of TCF12 can sensitize melanoma to BRAF inhibition both in vitro and in vivo. Overall, our results demonstrate that TCF12 promotes melanoma progression and can be a potential tumor therapeutic target.

## 1. Introduction

Melanoma is a malignant tumor that originates from normal epidermal melanocytes or pre-existing nevus cells [1]. The global incidence of melanoma has been steadily increasing in recent years, posing a significant health challenge [2]. Unfortunately, a major concern is the late-stage diagnosis of most patients, when invasion and metastasis have already occurred, leading to higher mortality rates [3]. The landscape of malignant melanoma treatment has witnessed a transformative shift with the emergence of targeted therapies directed against BRAF(V600E) mutation and immune checkpoint-blocking antibodies [4,5,6]. These groundbreaking advancements have substantially improved survival rates among patients with advanced-stage disease. Nevertheless, treatment responses remain heterogeneous [7,8]. A comprehensive understanding of the underlying mechanisms contributing to the heightened risk of recurrence holds tremendous potential for enhancing clinical management and optimizing patient outcomes through tailored surveillance and adjuvant treatment strategies.

BRAF(V600E) is the most prevalent mutation in melanoma, regulating tumor growth, invasion, and metastasis [9]. Among the diverse downstream pathways of BRAF(V600E), transcriptional regulation presents an essential one [10]. For example, BRAF(V600E) is known to directly enhance the stability and activity of microphthalmia-associated transcription factor (MITF), the master regulator of melanocyte development and differentiation, leading to increased cell proliferation, survival, and resistance to therapies [11,12]. Additionally, we have previously reported that BRAF(V600E) negatively regulates the expression and activity of the transcriptional coactivator PGC1α (peroxisome proliferator-activated receptor gamma coactivator 1-alpha), fine-tuning melanoma metabolism and transcriptional programs to balance tumor growth and metastatic spreading [13,14]. However, it is still not fully understood whether other novel transcriptional factors and programs act downstream of BRAF(V600E) in the regulation of melanoma progression.

Transcription factor 12 (TCF12), also known as HTF4 or HEB, is a member of the helix-loop-helix (HLH) protein family. It plays a crucial role in cell development and differentiation across various tissues, including skeletal muscle, neurons, mesenchymal tissues, and lymphocytes [15,16,17,18]. TCF12 can form homodimers or heterodimers with other members of the HLH family to activate gene expression [19]. Recent studies have highlighted the contributions of TCF12 to the progression of different tumor types, including colorectal, pancreatic, liver, and ovarian cancers [20,21,22,23]. Additionally, TCF12 expression has also been found to be associated with advanced tumor stages and poor prognosis in breast and lung cancers [24,25]. Interestingly, our early studies suggested that TCF12 may be a functional partner of PGC1α, likely playing a role in melanoma metastasis [13,14]. However, whether TCF12 regulates melanoma progression and, if yes, by what mechanism, remain elusive to date.

In this study, we explored the functional involvement and molecular mechanisms of TCF12 in melanoma. We found that the expression level of TCF12 is elevated as melanoma progresses, and high expression is strongly associated with poor survival outcomes in melanoma patients. Functionally, we found that TCF12 can enhance melanoma proliferation and metastasis, as well as sensitivity to BRAF(V600E)-targeted therapy. Mechanistically, we identified TGFB2 as a direct transcriptional target of TCF12, mediating its pro-tumorigenic effects. Collectively, our study supported the oncogenic functions of TCF12 in melanoma, revealing it as a potential target to improve the efficacy of BRAF(V600E)-targeted therapy.

## 2. Materials and Methods

### 2.1. Cell Lines and Cell Culture

The human melanoma cell line A375 and murine melanoma cell line YUMM1.7 utilized in this study were purchased from the American Type Culture Collection (ATCC). The human kidney cell line HEK293T was purchased from the Cell Bank of the Chinese Academy of Sciences. The cells were regularly tested for mycoplasma contamination using the MycoAlert Mycoplasma Detection Kit (Lonza, Basel, Switzerland) and verified by morphological observation. Cells were cultured in Dulbecco’s Modified Eagle’s Medium (DMEM, 11965084, Gibco, Billings, MT, USA) supplemented with 10% fetal bovine serum (FBS, 10099141, Gibco, Billings, MT, USA), 100 U/mL penicillin, and 100 mg/mL streptomycin (15140122, Gibco, Billings, MT, USA). They were kept at 37 °C in a humidified incubator containing 5% CO_2_. The medium was replaced every 2–3 days, and upon attaining 80–90% confluency, the cells underwent subculturing.

### 2.2. Reagents and Antibodies

The following primary antibodies were used for immunoblotting: TCF12/HEB (D2C10) rabbit mAb (Cell Signaling Technology, Danvers, MA, USA, dilution: 1:1000), TGFB2 rabbit pAb (A3640) (ABclonal, Woburn, MA, USA, dilution: 1:500), alpha tubulin monoclonal antibody (1E4C11)-HRP (Proteintech, Rosemont, IL, USA, dilution: 1:3000), and beta actin monoclonal antibody (7D2C10)-HRP (Proteintech, dilution: 1:3000). The following primary antibodies were used for immunohistochemical (IHC) staining: TCF12/HEB antibody (14419-1-AP) (Proteintech, dilution: 1:100), TGFB2 rabbit pAb (A3640) (ABclonal, dilution: 1:50), and anti-Ki-67 rabbit pAb (ab15580) (Abcam, dilution: 1:400). The following primary antibodies were used for immunoprecipitation: TCF12/HEB (D2C10), rabbit mAb (Cell Signaling Technology, 2 μL), and rabbit control IgG (AC005) (ABclonal, 2 μL).

The BRAF(V600E) inhibitor PLX4032 (#S1267) was purchased from Selleck Chemicals (Houston, TX, USA). The MEK inhibitor trametinib (#SD5973) was purchased from Beyotime (Shanghai, China). MG132 (ab141003) was purchased from Abcam (Cambridge, United Kingdom). Melanoma tissue microarray (DC-Mel21020) was purchased from Shaanxi Avila Biotechnology (Shaanxi, China).

### 2.3. RNA Interference

For TCF12 knockdown experiments, short hairpin RNA (shRNA) oligonucleotides were cloned into the lentiviral vector pLKO.1. Lentiviruses were produced in HEK293T cells by co-transfection with packaging vectors pMD2G and psPAX2 using Lipofectamine 3000 (L30000015, Invitrogen, Waltham, MA, USA) according to the manufacturer’s instructions. Lentivirus-containing supernatants were collected 48 h after transfection, filtered through a 0.45 µm filter, and used to infect A375 and YUMM1.7 melanoma cells. Polybrene (8 µg/mL) was added to enhance infection efficiency. Infected cells were selected with 2 μg/mL of puromycin for at least 4 days before subsequent experiments. The shRNA clones targeting TCF12 were as follows: shTCF12-1: 5′-CCATCCCATAATGCACCAATT-3′; shTCF12-2: 5′-GCTGTGATTATGGTGAACATA-3′; shTcf12-1: 5′-TGTATGTCACTGTGGCTAGT-3′; shTcf12-2: 5′-CAGTCTTGATTTCTGTTGGAAC-3′. siTgfb2-1: 5′-GACCCUACUUCAGAAUCGUTT-3′; siTgfb2-2: 5′-GAGGGAUCUUGGAUGGAAATT-3′.

### 2.4. Cell Proliferation and Colony Formation Assays

To evaluate cell proliferation, A375 and YUMM1.7 cells were seeded in 6-well plates at a density of 5 × 10^3^ cells per well in triplicate. The cell number was counted at the indicated time points using a hemocytometer. For drug treatment experiments, cells were exposed to either DMSO (vehicle control) or PLX4032 the day after seeding, followed by cell counting at the indicated time points.

To assess the clonogenic potential of cells, A375 and YUMM1.7 cells were seeded in 6-well plates at a density of 1 × 10^2^ cells per well in triplicate. After two weeks of incubation, colonies were fixed with 100% ethanol for 10 min and stained with a 0.5% crystal violet solution in 25% methanol for 20 min. Excess stain was washed with water, and the plates were air-dried before counting the colonies.

### 2.5. Migration and Invasion Assays

Cell migration was assessed using transwell chambers with an 8 µm pore size (Corning Life Science). YUMM1.7 EV/Tcf12 (1 × 10^4^), YUMM1.7 shScr/shTcf12 (2 × 10^4^), A375 EV/TCF12 (2 × 10^4^), or A375 shScr/shTCF12 (3 × 10^4^) cells were suspended in 0.1 mL of FBS-free medium and seeded into the upper chamber. The lower chamber was filled with a medium containing 10% FBS as a chemoattractant. Cells were incubated at 37 °C in a humidified incubator containing 5% CO_2_. After incubation, non-migrated cells in the upper chamber were gently removed with a cotton swab. Migrated cells attached to the lower surface of the membrane were fixed with 4% paraformaldehyde for 20 min and stained with a 0.5% crystal violet solution in 25% methanol for 20 min. The membrane was then rinsed with water, air-dried, and mounted onto a glass slide. Cells from three random fields were imaged under 10× or 20× magnification using a Nikon Ti-s inverted microscope, and the total number of migrated cells was quantified.

For the invasion assay, the upper chamber was coated with Matrigel (Corning) before seeding the cells. The subsequent steps were the same as the migration assay. Cells that had invaded through the Matrigel-coated membrane were fixed, stained, and quantified as described above.

### 2.6. Immunoblotting

A375 and YUMM1.7 cells underwent lysis in RIPA buffer (50 mM Tris-HCl pH 7.4, 150 mM NaCl, 1% NP-40, 0.5% sodium deoxycholate, 0.1% SDS, protease and phosphatase inhibitors). Whole-cell extracts were harvested, and protein concentration was determined using the BCA Protein Assay Kit (P0010, Beyotime).

Subsequently, equal amounts of protein were separated through sodium dodecyl sulfate-polyacrylamide gel electrophoresis (SDS-PAGE) and electro-transferred nitrocellulose or polyvinylidene fluoride (PVDF) membranes. To prevent nonspecific binding, the membranes were incubated in a blocking solution of 5% non-fat dry milk or bovine serum albumin (BSA) in Tris-buffered saline with 0.1% Tween 20 (TBST) for 1 h at room temperature. Following this, the membranes were incubated in a blocking solution containing the appropriate primary antibodies overnight at 4 °C.

After washing with TBST three times, the membranes were incubated in a blocking solution containing secondary antibodies conjugated by horseradish peroxidase (HRP) for 1 h at room temperature. The protein levels were visualized using an enhanced chemiluminescence (ECL) kit (Bio-Rad, Hercules, CA, USA), and images were captured using a chemiluminescence imaging system.

### 2.7. Quantitative Real-Time PCR

Total RNA was isolated from cells using the TRIzol reagent (Life Technologies, Carlsbad, CA, USA) according to the manufacturer’s instructions. A total of 2 μg of RNA was reverse transcribed into complementary DNA (cDNA) using the M-MLV reverse transcriptase (Takara, Kusatsu, Japan) and random primers. The cDNA was used as a template for real-time PCR analysis, which was carried out using SYBR Green PCR Master Mix (Takara) on a real-time PCR system. Experimental Ct values were normalized to the housekeeping genes, and relative mRNA expression was calculated using the 2^−ΔΔCT^ method. Each sample was analyzed in triplicate, and the mean Ct value was used for calculations. The primer sequences used for RT-qPCR are shown in Table S1.

### 2.8. Immunohistochemical (IHC) Staining

The tissue samples were prepared by fixing them in 10% buffered formalin for an overnight period, followed by preservation in 70% ethanol prior to being embedded in paraffin. They were then sectioned and stained with hematoxylin and eosin (H&E). The resulting paraffin sections (4 µm in thickness) were subjected to deparaffinization in xylene and sequentially rehydrated using a descending concentration of ethanol. For optimal antigen exposure, these sections were immersed in 0.1 M citrate buffer (pH 6.0) and brought to a boil either via microwave or water bath. Once cooling to room temperature, the sections underwent washing with phosphate-buffered saline (PBS). Endogenous peroxidase activity was neutralized using 3% hydrogen peroxide. To block non-specific interactions, sections were treated with 10% normal goat serum for 30 min at room temperature. The sections were then incubated with indicated antibodies at 4 °C overnight. After PBS washing, the sections were incubated with biotinylated secondary antibodies. This was followed by an incubation with streptavidin-horseradish peroxidase (HRP) conjugate using the mouse/rabbit two-step assay kit (Mouse/Rabbit Polymer Assay Detection System, ZSGB-Bio, Beijing, China). The immunoreactivity was visualized using 3,3′-diaminobenzidine (DAB) as the chromogen, and the sections were counterstained with hematoxylin. All tissue slides were photographed using a Leica DM2000 upright microscope. The immunostaining was scored based on the positive percentage and staining intensity of positively staining cells by a pathologist blinded to the experimental conditions.

### 2.9. Animal Experiments

All animal experiments were designed and conducted following the protocol approved by the Medical Experimental Animal Care Commission of Zhejiang University (#ZJU20220217). Six- to eight-week-old male C57BL/6 mice (purchased from Shanghai Slack Laboratory Animal Co., Ltd., Shanghai, China) were used for this study. Mice were housed in a controlled environment under a 12 h dark/12 h light cycle, with food and water provided ad libitum.

Before the injection, YUMM1.7 cells were detached using 0.5 mM EDTA in PBS and washed with 1× PBS. A total of 1 × 10^5^ YUMM1.7 melanoma cells with vector control or Tcf12 overexpression were injected subcutaneously into the flanks of mice in 0.1 mL of PBS, and the tumor development was monitored every other day. Tumor volume was calculated based on the equation V = (width (in mm)^2^ × length (in mm))/2. For melanoma cells with Tcf12 knockdown, 2 × 10^5^ cells were used for subcutaneous injection.

For the drug administration experiment, tumor-bearing mice were given 20 mg/kg PLX4032 (in 10% NMP and 90% PEG) or vehicle via oral administration every other day after subcutaneous tumors could be detected.

To establish a tumor lung metastasis model, a total of 1 or 2 × 10^5^ YUMM1.7 melanoma cells with Tcf12 overexpression or knockdown were injected into the tail veins of C57BL/6 mice in 0.2 mL of PBS. Lung tissues were harvested 3–4 weeks after injection. The survival time of the mice was recorded to generate the survival curve.

For histological analysis, subcutaneous tumors and lungs from mice were collected, fixed, paraffin-embedded, and sectioned for hematoxylin and eosin (H&E) or immunohistochemical (IHC) staining.

### 2.10. RNA-Sequencing Analysis

RNA was harvested from YUMM1.7 cells of both the stable negative control (shScr) and Tcf12 knockdown (shTcf12-1) utilizing the TRIzol reagent (Life Technologies). The total RNA concentration and purity were validated by a NanoDrop™ One Microvolume UV–vis spectrophotometer (Thermo Scientific, Waltham, MA, USA).

Novogene Co., Ltd. (Beijing, China) conducted the RNA-seq analysis. Briefly, the RNA quality was verified by an Agilent 2100 bioanalyzer (Agilent Technologies, Santa Clara, CA, USA). Oligo-dT magnetic beads facilitated the purification of mRNA with poly-A tails, which were subsequently fragmented into small pieces through divalent cations at a heightened temperature. These fragments then underwent first-strand cDNA synthesis with the aid of random hexamer primers and reverse transcriptase. This was followed by a second-strand cDNA synthesis using DNA polymerase I and RNase H. The double-stranded cDNA fragments were subjected to end repair, A-tailing, adapter ligation, and PCR amplification. The final library quantification was carried out with the Qubit 2.0 fluorometer (Life Technologies), and an Agilent 2100 bioanalyzer (Agilent Technologies) verified the insert size and calculated the library concentration.

The resulting cDNA library was sequenced by an Illumina NovaSeq 6000 platform, producing 150 bp paired-end reads. Raw read processing eliminated low-quality reads and adapter sequence contaminants. The refined, high-quality clean reads were then mapped to the reference genome via HISAT2. Transcript abundance estimation and differential expression evaluations were conducted utilizing StringTie and DESeq2, respectively. Differentially expressed genes (DEGs) were identified based on criteria: a false discovery rate (FDR) < 0.05 and an absolute fold change ≥ 2.

The DEGs underwent a functional enrichment analysis utilizing the Gene Ontology (GO) and Kyoto Encyclopedia of Genes and Genomes (KEGG) pathway databases. The significance of enrichment was determined using a corrected *p*-value < 0.05.

### 2.11. Chromatin Immunoprecipitation (ChIP)

YUMM1.7 melanoma cells with Tcf12 overexpression or Tcf12 knockdown were crosslinked by 1% paraformaldehyde, and then 10% glycine was added to quench untreated paraformaldehyde. Cells from each dish were scraped into PBS and centrifuged at 800 rpm for 5 min. The pellets were resuspended in swelling buffer for 10 min of rotation at 4 °C, centrifuged at 1000 rpm for 15 min at 4 °C, and resuspended in SDS lysis buffer. Following sonication, equal amounts of lysates were incubated with IgG or TCF12 antibodies overnight at 4 °C.

The lysates were then incubated with protein A/G-magnetic beads (Thermo Scientific) for 2 h to precipitate the antibody-bound chromatin. The beads were washed sequentially with low-salt buffer, high-salt buffer, LiCl buffer, and TE buffer to remove any nonspecific interactions. The immunoprecipitated chromatin was then eluted from the beads and reverse crosslinked at 65 °C overnight to obtain the DNA.

After treatment with RNase A and Proteinase K to degrade RNA and protein contaminants, DNA was purified using a PCR purification kit (Qiagen, Venlo, The Netherlands) according to the manufacturer’s instructions. The purified DNA was then subjected to quantitative real-time PCR (qPCR) using SYBR Green PCR Master Mix (Takara) to analyze the enrichment of specific genomic regions. The primer sequences used for qPCR are shown in Table S2.

### 2.12. Luciferase Reporter Assay

The fragment of the Tgfb2 promoter was inserted into the pGL3 luciferase reporter vector, which was synthesized by Miaoling Biotechnology, Heze, China. The fragments containing mutated binding sites were constructed using the Fast Mutagenesis System Kit (TransGene Biotech, Beijing, China). YUMM1.7 cells were seeded in 24-well plates and co-transfected with pGL3 vectors containing either the wild-type or mutated Tgfb2 promoter, pRL-TK Renilla luciferase vector (for normalization), and either the Tcf12 overexpression or Tcf12 knockdown plasmid using Lipofectamine 3000 (Invitrogen) according to the manufacturer’s instructions. Forty-eight hours after transfection, cells were harvested and lysed using a passive lysis buffer. The luciferase activity of both firefly and Renilla luciferases was analyzed using a dual-luciferase reporter assay system (Promega, Madison, WI, USA) according to the manufacturer’s instructions. The total light intensity was measured using a VarioskanTM LUX microplate reader (Thermo Scientific). The firefly luciferase activity was normalized to Renilla luciferase activity to account for any differences in transfection efficiency. The relative luciferase activity was calculated by comparing the normalized luciferase activity of cells transfected with the Tcf12 overexpression plasmid to that of cells transfected with the control vector.

### 2.13. Statistical Analysis

No statistical methods were used to predetermine sample size for in vivo and in vitro experiments, but at least three biologically independent samples were used per experimental group and condition. The data were presented as mean ± standard deviation (SD). Statistical analysis was conducted employing the GraphPad Prism 9.5 software. Comparisons involving two sets were made through a two-tailed unpaired Student’s t-test. In assessing survival, we utilized the Kaplan–Meier approach to generate survival curves, and differences were calculated by a *p*-value below 0.05, which was deemed to indicate statistical significance.

## 3. Results

### 3.1. TCF12 Expression Is Positively Correlated with Poor Prognosis in Melanoma Patients

**Figure 1:**
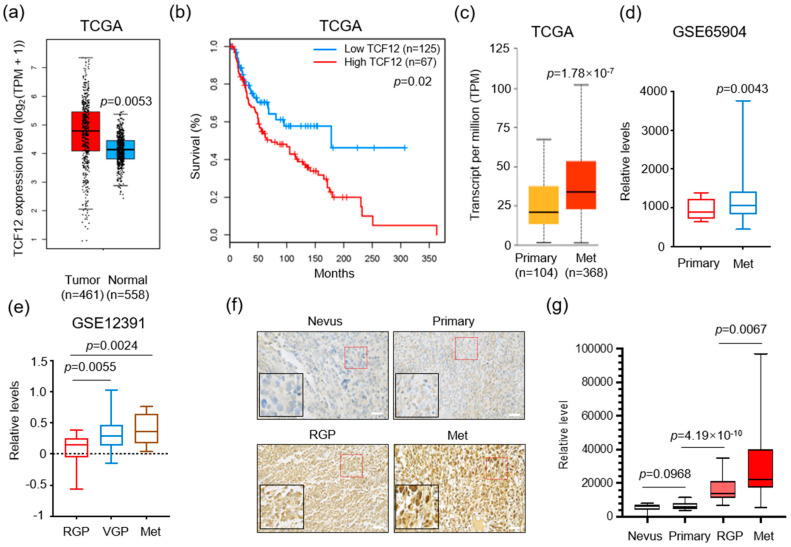
TCF12 expression correlates with melanoma progression and poor patient prognosis: (**a**) Boxplot illustrating the expression of TCF12 in melanoma tissues compared with normal skin samples, data derived from The Cancer Genome Atlas (TCGA) database; (**b**) Kaplan−Meier survival analysis comparing overall survival in melanoma patients grouped by low and high TCF12 expression levels; (**c**,**d**) analysis of relative TCF12 expression in primary melanomas compared to metastatic tissues (Met) using TCGA database (**c**) and the GSE65904 dataset (**d**); (**e**) comparative analysis of TCF12 expression levels across different stages of melanoma: radial growth phase (RGP), vertical growth phase (VGP), and metastatic phase (Met). Statistical significance is based on comparison with the RGP group; (**f**,**g**) representative images of immunohistochemistry (IHC) staining for TCF12 in melanoma tissue samples (red square, 200×; black square, 400×) (**f**), and corresponding histological analysis quantifying TCF12 protein levels (**g**). Scale bar: 100 μm. Statistical significance is based on comparison indicated in the graphs.

To examine the expression of TCF12 in melanoma patients, we utilized the GEPIA (Gene Expression Profiling Interactive Analysis) online platform to profile TCF12 mRNA levels in melanoma patients and normal tissues [26]. The results revealed that melanoma tissues had a notably elevated expression of TCF12 compared to adjacent normal skins (Figure 1a). Moreover, patients with an increased TCF12 expression level showed reduced overall survival (Figure 1b), suggesting a role for TCF12 in melanoma progression. Indeed, we confirmed that the expression of TCF12 increases as disease progresses, as evidenced by higher levels in metastatic tissues than in primary melanomas (Figure 1c,d). Interestingly, we also found that even within the primary melanomas, the vertical growth phase (VGP) tumors, which have penetrated deeper into the skin layers, showed a higher level of TCF12 compared to tumors in the radial growth phase (RGP), which represent the very early stage of melanoma (Figure 1e). To further validate the findings from the public dataset, we performed TCF12 immunohistochemistry (IHC) on a melanoma tissue array. In line with the bioinformatic analyses, our results demonstrated that the TCF12 protein was upregulated in melanoma and elevated progressively as the tumor advanced (Figure 1f,g). These findings suggest a potential functional role for TCF12 in melanoma progression and metastasis.

### 3.2. TCF12 Enhances Melanoma Cell Proliferation In Vitro and Tumorigenicity In Vivo

**Figure 2:**
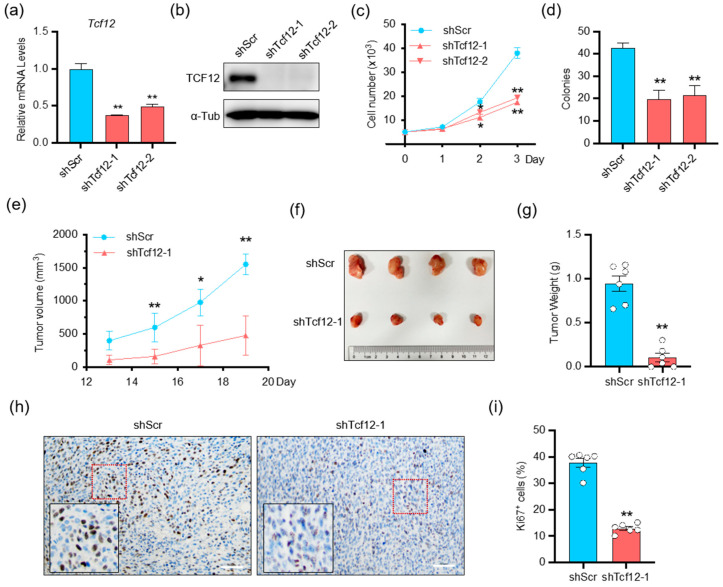
TCF12 enhances melanoma cell proliferation in vitro and tumorigenicity in vivo: (**a**,**b**) qPCR (**a**) and immunoblot (**b**) analysis of TCF12 level in YUMM1.7 cell lines after TCF12 knockdown. shScr: control shRNA, shTcf12-1/2: mouse TCF12-specific shRNA; (**c**,**d**) cell proliferation (**c**) and colony formation capability (**d**) in the TCF12 knockdown cells as compared to control cells; (**e**) tumor growth curves in mice injected with YUMM1.7 cells with TCF12 knockdown, n = 6 mice per group; (**f**) representative tumor image from control (shScr) and TCF12-knockdown (shTcf12-1) mice; (**g**) tumor weights comparison between control and TCF12 knockdown groups; (**h**,**i**) Ki67 immunohistochemistry staining representative images (red square, 200×; black square, 400×) (**h**) and subsequent analysis (**i**) in tumor tissues. Scale bar: 50 μm. Statistical significance is based on comparison with shScr group. * *p* < 0.05, ** *p* < 0.01.

To explore the biological function of TCF12 in melanoma, we first generated stable TCF12 knockdown in both human and mouse melanoma cells (A375 and YUMM1.7) (Figure 2a,b and Figure S1a,b). Proliferation and colony formation assays revealed that TCF12 knockdown led to the inhibition of cell growth and reduced colony formation capacity (Figure 2c,d and Figure S1c,d). Subsequently, we carried out ectopic TCF12 expression experiments (Figures S2a,b and S1e,f) and found that TCF12 overexpression promoted melanoma cell proliferation and colony formation (Figures S2c,d and S1g,h). Next, we tested whether TCF12 affected the tumorigenesis of melanoma in vivo in an immunocompetent background. Knockdown of TCF12 significantly impaired the growth of YUMM1.7 tumors, as evidenced by slower tumor growth kinetics (Figure 2e) and smaller tumor size at the endpoint (Figure 2f,g), in line with a lower proliferation index by Ki67 IHC (Figure 2h,i). On the other hand, overexpression of TCF12 greatly accelerated YUMM1.7 tumor progression (Figure S2e–g), consistent with more Ki67+ cells in the tumor tissues (Figure 2h,i). Collectively, these results indicate that TCF12 has an oncogenic function and promotes melanoma tumorigenesis both in vitro and in vivo.

### 3.3. TCF12 Promotes Melanoma Cell Migration, Invasion In Vitro and Metastasis In Vivo

**Figure 3:**
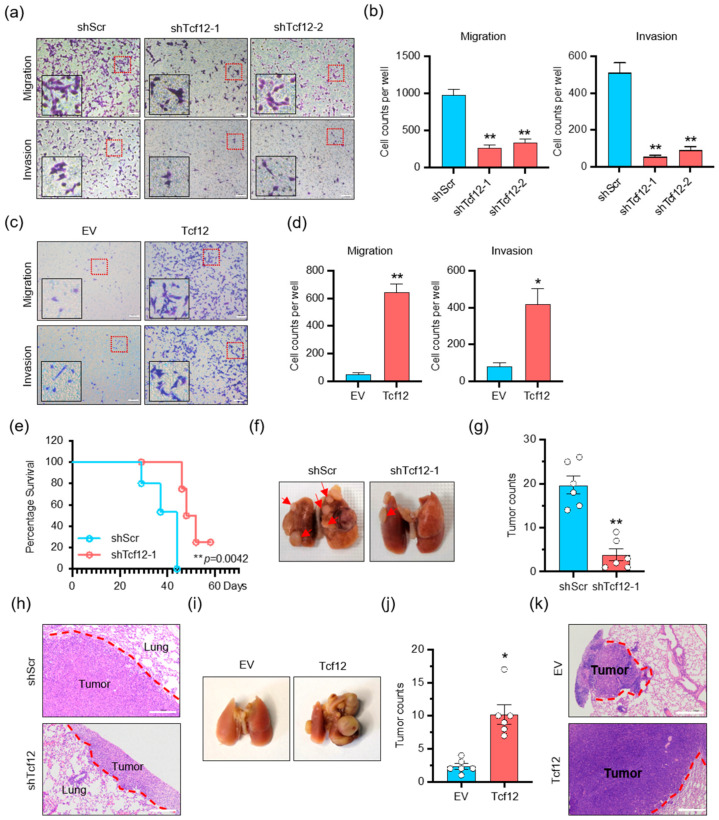
TCF12 promotes melanoma cell migration, invasion in vitro and metastasis in vivo: (**a**,**b**) The representative images (red square, 200×; black square, 400×) (**a**) and cell count analysis (**b**) of transwell migration and Matrigel invasion assays of YUMM1.7 cells after TCF12 knockdown. shScr: control shRNA, shTcf12-1/2: mouse TCF12-specific shRNA; (**c**,**d**) the representative images (red square, 200×; black square, 400×) (**c**) and cell count analysis (**d**) of transwell migration and Matrigel invasion assays of YUMM1.7 cells following TCF12 overexpression. EV: empty vector expression; Tcf12: mouse TCF12 overexpression; (**e**) Kaplan–Meier survival curves of mice after tail-vein injection of TCF12 knockdown YUMM1.7 cells, n = 6 per group; (**f**–**h**) representative images of lung metastasis (**f**), hematoxylin and eosin (H&E) staining of lung sections (**h**), and the number of tumor nodules (**g**) in mice injected via tail vein with control (shScr) and TCF12 knockdown (shTcf12) YUMM1.7 cells. Scale bar: 100 μm. Representative images of lung metastasis in mice tail-vein injected with control (shScr) and TCF12 knockdown (shTcf12) YUMM1.7 cells; (**i**–**k**) representative images of lung metastasis (**i**), H&E staining of lung sections (**k**), and the number of tumor nodules (**j**) in mice tail-vein injected with control (EV) and TCF12 overexpression (Tcf12) YUMM1.7 cells, n = 6 mice per group. Scale bar: 100 μm in H&E staining. Statistical significance is based on comparison with shScr group or EV group. * *p* < 0.05, ** *p* < 0.01.

We continued to investigate whether TCF12 in melanoma affected cell migration and metastasis. In vitro transwell migration and Matrigel invasion assays showed that TCF12 knockdown reduced the migration and invasion of melanoma cells (Figure 3a,b and Figure S3a,b), whereas TCF12 overexpression greatly enhanced their migratory and invasive capacities (Figure 3c,d and Figure S3c,d). Similarly, knockdown of TCF12 significantly compromised the metastatic outgrowth of melanoma cells in mice after tail-vein injection, as revealed by improved animal survival (Figure 3e) and fewer macroscopic and microscopic tumor nodules in the lung (Figure 3f–h). In contrast, overexpression of TCF12 in melanoma enabled the cells to establish larger and larger tumor nodules in the lung upon tail-vein injection (Figure 3i–k). Taken together, these findings indicate that TCF12 promotes melanoma metastasis in vitro and in vivo.

### 3.4. TGFB2 Is a Direct Downstream Target Gene of TCF12

**Figure 4:**
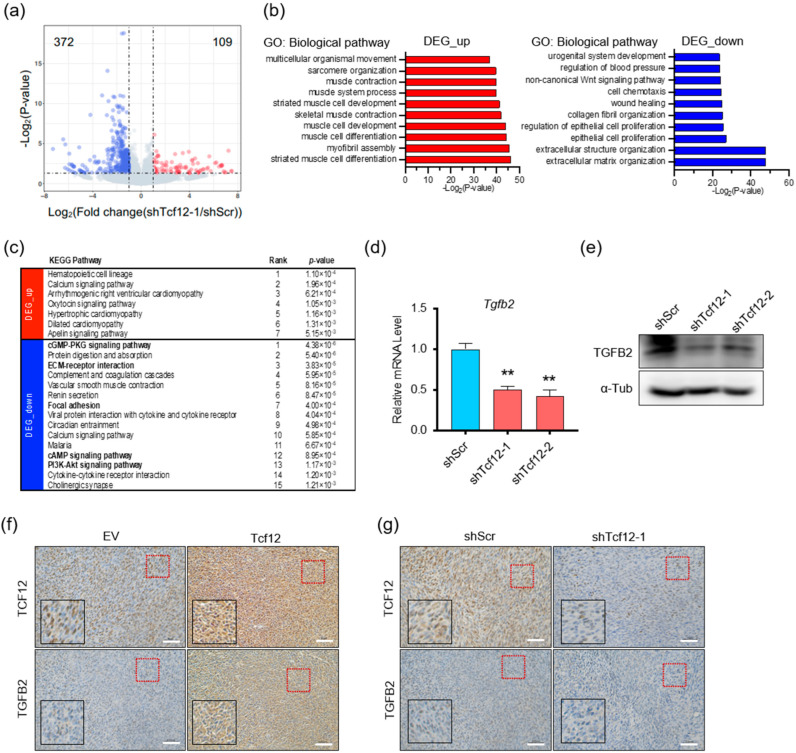
TGFB2 is a downstream target of TCF12: (**a**) Volcano plot of differentially expressed genes in YUMM1.7 cells with TCF12 knockdown (shTcf12-1) compared to control (shScr), analyzed by RNA-seq (blue, genes down-regulated; red, genes up-regulated); (**b**,**c**) gene ontology (GO) enrichment (**b**) and KEGG pathway (**c**) analysis for biological processes of the differentially expressed genes upon TCF12 knockdown; (**d**,**e**) qPCR (**d**) and immunoblot (**e**) analysis of TGFB2 expression in YUMM1.7 cells upon TCF12 knockdown; (**f**,**g**) representative images of TGFB2 IHC staining in mouse melanoma tissues with TCF12 knockdown (**g**) or TCF12 overexpression (**f**) (red square, 200×; black square, 400×). Scale bar: 50 μm. Statistical significance is based on comparison with shScr group. ** *p* < 0.01.

To elucidate the key molecules involved in TCF12-induced proliferation and metastasis in melanoma, we performed RNA-seq analysis on YUMM1.7 cells with scramble control or TCF12 knockdown. The volcano plot showed that 372 and 109 genes were significantly downregulated and upregulated upon TCF12 depletion in melanoma cells, respectively (Figure 4a). Gene ontology enrichment analysis found that the upregulated genes were enriched in muscle developmental and functional programs, while the downregulated genes were enriched in cell mobility, extracellular matrix organization, and proliferation (Figure 4b). Consistently, KEGG pathway analysis also revealed similar enrichment (Figure 4c). Particularly, the detection of ECM–receptor interaction, focal adhesion, cGMP, cAMP, and PI3K-AKT signaling pathways among the top lists for downregulated genes was consistent with our observation that knockdown of TCF12 impaired melanoma proliferation and metastasis. Subsequently, we screened a set of melanoma-related target genes with TCF12 binding sites by RT-qPCR (Figure S4 and Table S3) and verified that TCF12 knockdown significantly decreased *Tgfb2* transcription (Figure 4d), leading to reduced protein expression (Figure 4e). The uncropped Western blots are shown in Figure S5. We further employed IHC on the matched mouse melanoma tissues to validate that TCF12 depletion decreased TGFB2 protein while TCF12 overexpression boosted TGFB2 levels (Figure 4f,g).

**Figure 5:**
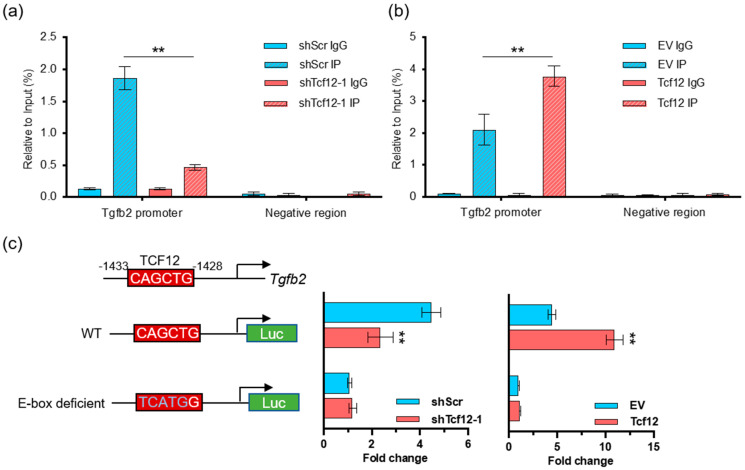
TCF12 directly binds to and activates the *Tgfb2* promoter: (**a**) Chromatin immunoprecipitation (ChIP) coupled with qPCR analysis showing the binding of TCF12 to the *Tgfb2* promoter in YUMM1.7 cells, which is decreased upon TCF12 knockdown; (**b**) ChIP−qPCR analysis showing the increased binding of TCF12 to the *Tgfb2* promoter in YUMM1.7 cells upon TCF12 overexpression; (**c**) the schematic diagrams show the *Tgfb2* promoter−driven luciferase reporters with either wild-type (WT) or E-box deficient sequences. Luciferase activity was measured and displayed as fold change. Statistical significance is based on comparison with the control group (shScr or EV). ** *p* < 0.01.

To determine whether TCF12 can directly modulate the transcription of TGFB2, chromatin immunoprecipitation (ChIP) coupled with qPCR was performed in melanoma cells. The results showed that TCF12 can occupy the *Tgfb2* promoter, which was significantly decreased upon TCF12 depletion (Figure 5a) but largely enhanced by TCF12 overexpression (Figure 5b). To further test whether the TCF12 binding is active, we constructed *Tgfb2* promoter-driven luciferase reporters with either wild-type or E-box-deficient sequences (Figure 5c). Luciferase assays showed that knockdown of TCF12 significantly impaired the reporter activity, while overexpression of TCF12 boosted the signal; however, depletion of the E-box, which mediates TCF12 binding, completely abolished the reporter activity (Figure 5c). In summary, we identified that TCF12 can directly bind to the *Tgfb2* promoter and activate its expression in melanoma.

### 3.5. TGFB2 Is Essential for TCF12-Induced Cell Proliferation, Migration and Invasion In Vitro

**Figure 6:**
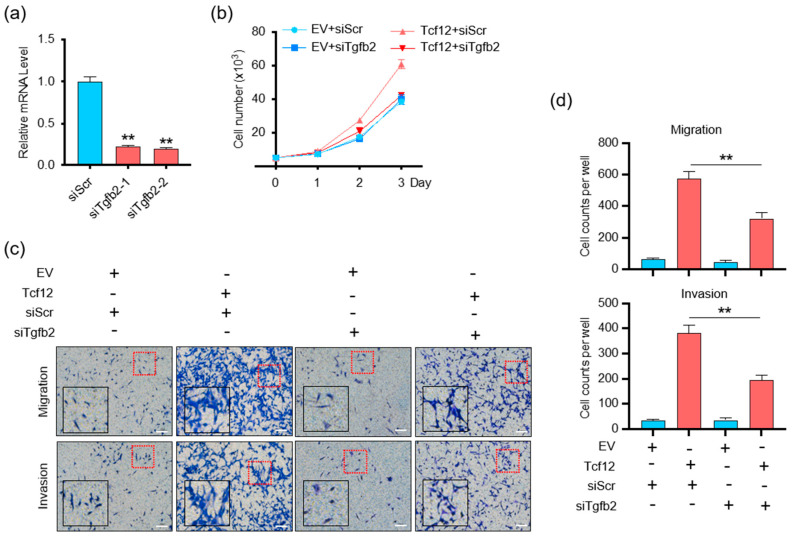
TGFB2 is essential for TCF12−induced cell proliferation, migration, and invasion in vitro: (**a**) qPCR analysis of TGFB2 level in YUMM1.7 cell lines after TGFB2 knockdown. siScr: control siRNA, siTgfb2-1/2: TGFB2 knockdown siRNA; (**b**) cell proliferation analysis of YUMM1.7 cells with TGFB2 knockdown, TCF12 overexpression, or their combination; (**c**,**d**) representative images (red square, 200×; black square, 400×) (**c**) and cell count analysis (**d**) of transwell migration and Matrigel invasion assays in YUMM1.7 cells with TGFB2 knockdown, TCF12 overexpression, or their combination. EV: empty vector expression, Tcf12: TCF12 overexpression, siScr: control siRNA, siTgfb2: TGFB2−specific siRNA. Scale bar: 100 μm. Statistical significance is based on comparison with control (Tcf12 + siScr) group. ** *p* < 0.01.

Given the critical functions of the TGF-β pathway in cancer biology [27,28,29,30], we speculated whether TGFB2 mediates the tumorigenic activity of TCF12 in melanoma. We found that, despite the fact that depletion of TGFB2 on its own did not alter melanoma proliferation, it was able to block the growth advantage conferred by TCF12 overexpression (Figure 6a,b). Similarly, TGFB2 knockdown significantly suppressed the migratory and invasive capabilities induced by TCF12 overexpression (Figure 6c,d). Together, we confirmed that TGFB2 was essential for TCF12 to induce melanoma proliferation, migration, and invasion.

### 3.6. Depletion of TCF12 Sensitizes Melanoma to BRAF Inhibition

**Figure 7:**
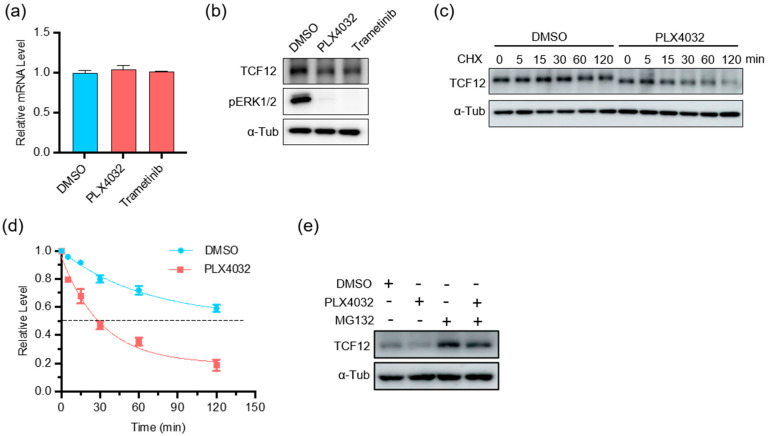
MAPK pathway inhibition reduces TCF12 protein stability: (**a**) qPCR analysis of TCF12 transcript in A375 cells treated with PLX4032 or trametinib for 24 h; (**b**) immunoblot analysis of TCF12 protein in A375 cells treated with PLX4032 or trametinib for 24 h. α-Tub: α-tubulin as internal control; (**c**,**d**) TCF12 protein stability analysis in A375 cells treated with PLX4032 for different time points. CHX: cycloheximide; (**e**) immunoblot analysis of TCF12 protein in A375 cells treated with PLX4032 alone or in combination with proteasome inhibitor MG132.

BRAF mutations represent the most prevalent oncogenic event in melanoma, resulting in constitutive activation of the BRAF-MEK-ERK MAPK pathway [31,32,33]. To test whether there is any correlation between BRAF signaling and TCF12, we treated BRAF-mutant A375 cells with the BRAF(V600E) inhibitor PLX4032 or the MEK inhibitor trametinib. We found that inhibition of the MAPK pathway did not alter the transcript of TCF12 (Figure 7a), but reduced the expression level of TCF12 protein (Figure 7b), suggesting a post-transcriptional regulation. We then measured the TCF12 protein stability in melanoma cells with or without BRAF inhibition and found that suppression of the BRAF pathway greatly reduced TCF12 protein stability (Figure 7c,d). We further found that the BRAF inhibition-facilitated TCF12 degradation was mediated by the proteosome, as the concurrent addition of the proteosome inhibitor MG132 markedly prevented the reduction of TCF12 protein by PLX4032 (Figure 7e).

**Figure 8:**
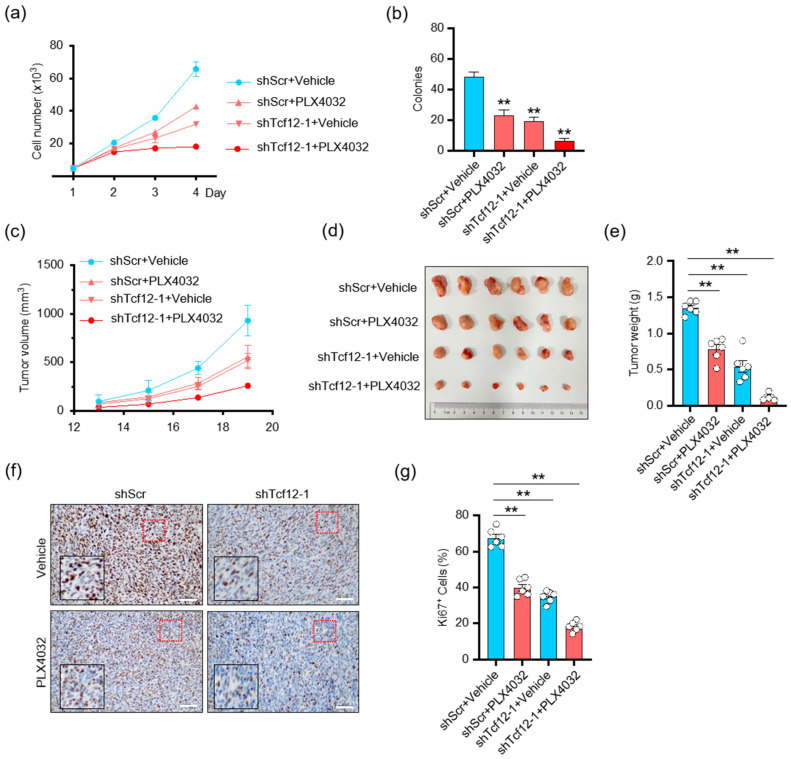
Depletion of TCF12 sensitizes melanoma to BRAF inhibition: (**a**,**b**) Cell proliferation (**a**) and colony formation (**b**) analysis of cells with TCF12 knockdown, PLX4032 treatment, or their combination; (**c**) tumor growth curves in mice injected cells with TCF12 knockdown and treated with PLX4032, n = 6 mice per group; (**c**,**g**) tumor growth curves (**c**), representative tumor image (**d**), tumor weights (**e**), representative images of Ki67 IHC (red square, 200×; black square, 400×) (**f**) and subsequent analysis (**g**) from control (shScr + vehicle), PLX4032 treatment (shScr + PLX4032), TCF12 knockdown (shTcf12-1 + vehicle), and their combination (shTcf12-1 + PLX4032) groups. Scale bar: 50 μm. Statistical significance is based on comparison with control group. ** *p* < 0.01.

Given that the BRAF pathway regulates TCF12 stability, we speculated whether TCF12 is involved in the sensitivity to BRAF-targeted therapy. We found that depletion of TCF12 substantially reduced melanoma proliferation in response to PLX4032, as measured by the cell growth curve (Figure 8a) and colony formation (Figure 8b). Consistently, the tumors with TCF12 deletion treated with PLX4032 showed the slowest growth kinetics (Figure 8c–e) and contained the smallest percentage of Ki67+ proliferating cells (Figure 8f,g). These data support the hypothesis that depletion of TCF12 sensitizes mutant melanoma to BRAF inhibitor therapy.

## 4. Discussion

**Figure 9:**
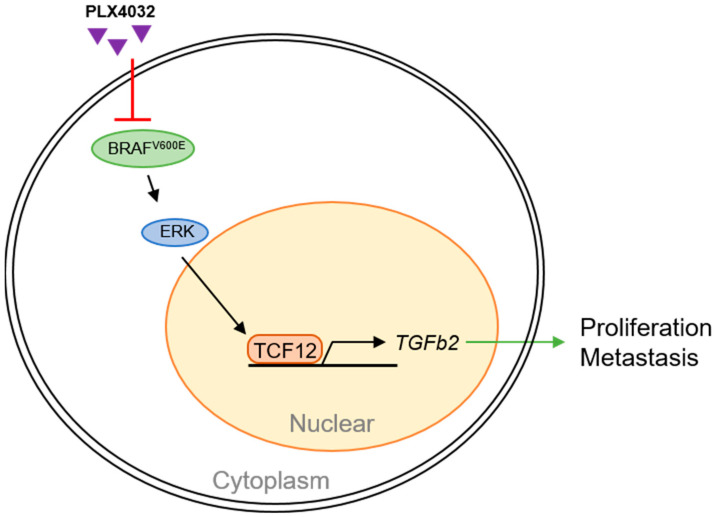
Schematic summary of TCF12 functions in melanoma pathogenesis.

Our findings shed light on a novel aspect of melanoma pathogenesis, uncovering the oncogenic functions of TCF12 and its regulatory mechanism within the disease (Figure 9). We found that TCF12 is upregulated in melanoma, and high expression is correlated with disease progression and a poorer prognosis. TCF12 enhances melanoma cell proliferation, metastasis, and sensitivity to BRAF(V600E)-targeted therapy. Importantly, we discovered that TCF12 exerts its oncogenic effects partly through transcriptional activation of TGFB2.

Melanoma exhibits a significant burden of genetic alterations that can regulate a myriad of biological processes, including cell proliferation, survival, differentiation, migration, and metastasis [34,35]. Mutations in BRAF, specifically the V600E variant, are the most common genetic alterations in melanoma, occurring in approximately 50% of cases [36]. Our study suggests that TCF12 is a new player in this intricate network of genetic interactions that contribute to melanoma progression. Moreover, our results indicate that the interaction between TCF12 and the BRAF pathway is crucial in mediating the tumor’s response to BRAF-targeted therapy.

The TGF-β signaling pathway has well-documented roles in tumor progression, promoting epithelial-to-mesenchymal transition, invasion, and metastasis in several cancers [37,38]. In melanoma, previous studies have reported a paradoxical role of TGF-β signaling [39]. On one hand, TGF-β has been shown to inhibit melanoma initiation by suppressing cell proliferation [40]. On the other hand, it promotes later stages of tumor progression by enhancing invasion and metastasis [41]. TGF-β protein is predominantly found in active melanocytes, whereas quiescent melanocytes exhibit minimal to non-existent TGF-β levels [42]. Typical melanocytes predominantly express TGFB1 and TGFB3. However, TGFB2 expression incrementally rises from initial to metastatic melanoma phases, suggesting its potential role in melanoma’s malignant evolution [43]. Notably, only a fraction of melanoma patients displays signs of TGFB2 reduction, hinting that its presence is not a primary event in melanoma development but rather associated with tumor progression [44]. Our finding that TGFB2 is a direct transcriptional target of TCF12 suggests a mechanism by which TCF12 contributes to melanoma progression. However, further studies are needed to fully elucidate the functional roles of TGFB2 in this context and whether its effects are context dependent.

Interestingly, we observed that TCF12 protein expression is regulated post-transcriptionally by the BRAF/MEK/ERK pathway. This observation implies a potential feedback loop wherein BRAF mutations upregulate TCF12, which in turn promotes tumor progression. Furthermore, we found that TCF12 depletion sensitizes melanoma cells to BRAF inhibition, suggesting that TCF12 may represent a potential therapeutic target for enhancing the efficacy of current BRAF-targeted therapies. This is of great clinical significance given the emergence of drug resistance as a significant problem in the treatment of BRAF-mutated melanomas.

We believe our findings offer promising avenues for future research and add to the growing body of knowledge that will hopefully lead to improved melanoma patient outcomes. However, the translation of these findings into clinical applications will require further investigations, including preclinical studies and potentially clinical trials, to validate the efficacy and safety of targeting TCF12 in the treatment of melanoma.

## 5. Conclusions

Our study provides new insights into the molecular mechanisms underlying melanoma progression and reveals a potential therapeutic target for melanoma treatment.

## Data Availability

The data presented in this study are available in the paper and/or the supplementary materials. Additional data related to this paper are available on request from the corresponding author.

## Supplementary Materials

The following supporting information can be downloaded at: https://www.mdpi.com/article/10.3390/cancers15184505/s1, Figure S1: TCF12 enhances melanoma A375 cell proliferation in vitro; Figure S2: Overexpression of TCF12 enhances melanoma cell proliferation in vitro and tumorigenicity in vivo; Figure S3: Overexpression of TCF12 promotes melanoma cell migration and invasion in vitro; Figure S4: Analysis of potential melanoma-related target genes of TCF12; Figure S5: The original western blot figures; Table S1: The primer sequences used for RT-qPCR; Table S2: The primer sequence used for ChIP-qPCR; Table S3: Differentially expressed gene after TCF12 knockdown.
