# Supplementary material for: TCF12 Activates TGFB2 Expression to Promote the Malignant Progression of Melanoma
Source: Cancers (Basel). 2023 Sep 11;15(18):4505. doi: 10.3390/cancers15184505 (PMC10527220; doi:10.3390/cancers15184505)
Supplement: Supplementary file 1 [file cancers-15-04505-s001.zip › Figure S1.pdf]

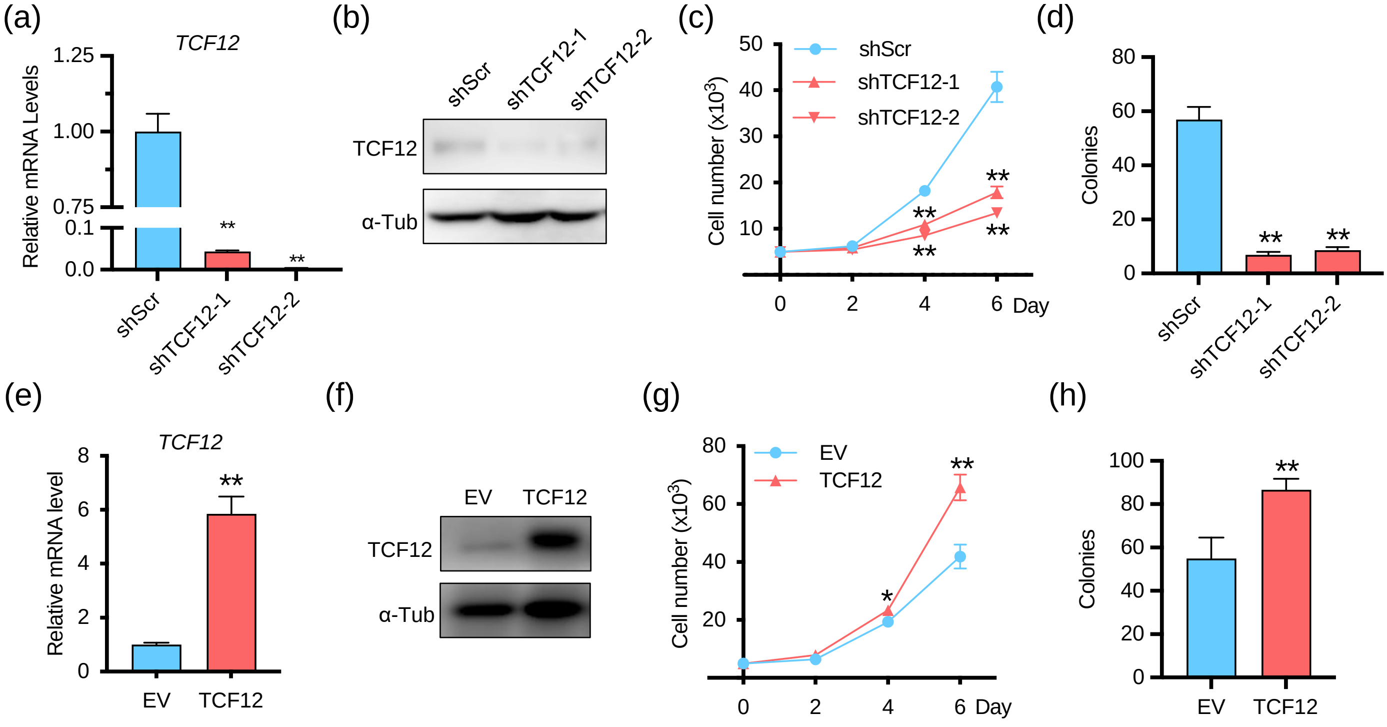

Figure S1. TCF12 enhances melanoma A375 cell proliferation *in vitro*: (**a**,**b**) qPCR (**a**) and immunoblot (**b**) analysis of TCF12 level in A375 cell lines after TCF12 knockdown. shScr: control shRNA, shTCF12-1/2: human TCF12 specific shRNA. α-Tub: α-tubulin as internal control; (**c**,**d**) Analysis of cell proliferation (**c**) and colony formation capability (**d**) in the TCF12 knockdown cells as compared to control cells; (**e**,**f**) qPCR (**e**) and immunoblot (**f**) analysis of TCF12 level in A375 cell lines after TCF12 overexpression. EV: empty vector expression, TCF12: human TCF12 plasmid overexpression; (**g**,**h**) Examination of cell proliferation (**g**) and colony formation capability (**h**) in the TCF12 overexpression cells as compared to control cells. Statistical significance is based on comparison with shScr group or EV group. * $p < 0.05$, ** $p < 0.01$.
