# Supplementary material for: TCF12 Activates TGFB2 Expression to Promote the Malignant Progression of Melanoma
Source: Cancers (Basel). 2023 Sep 11;15(18):4505. doi: 10.3390/cancers15184505 (PMC10527220; doi:10.3390/cancers15184505)
Supplement: Supplementary file 1 [file cancers-15-04505-s001.zip › Figure S2.pdf]

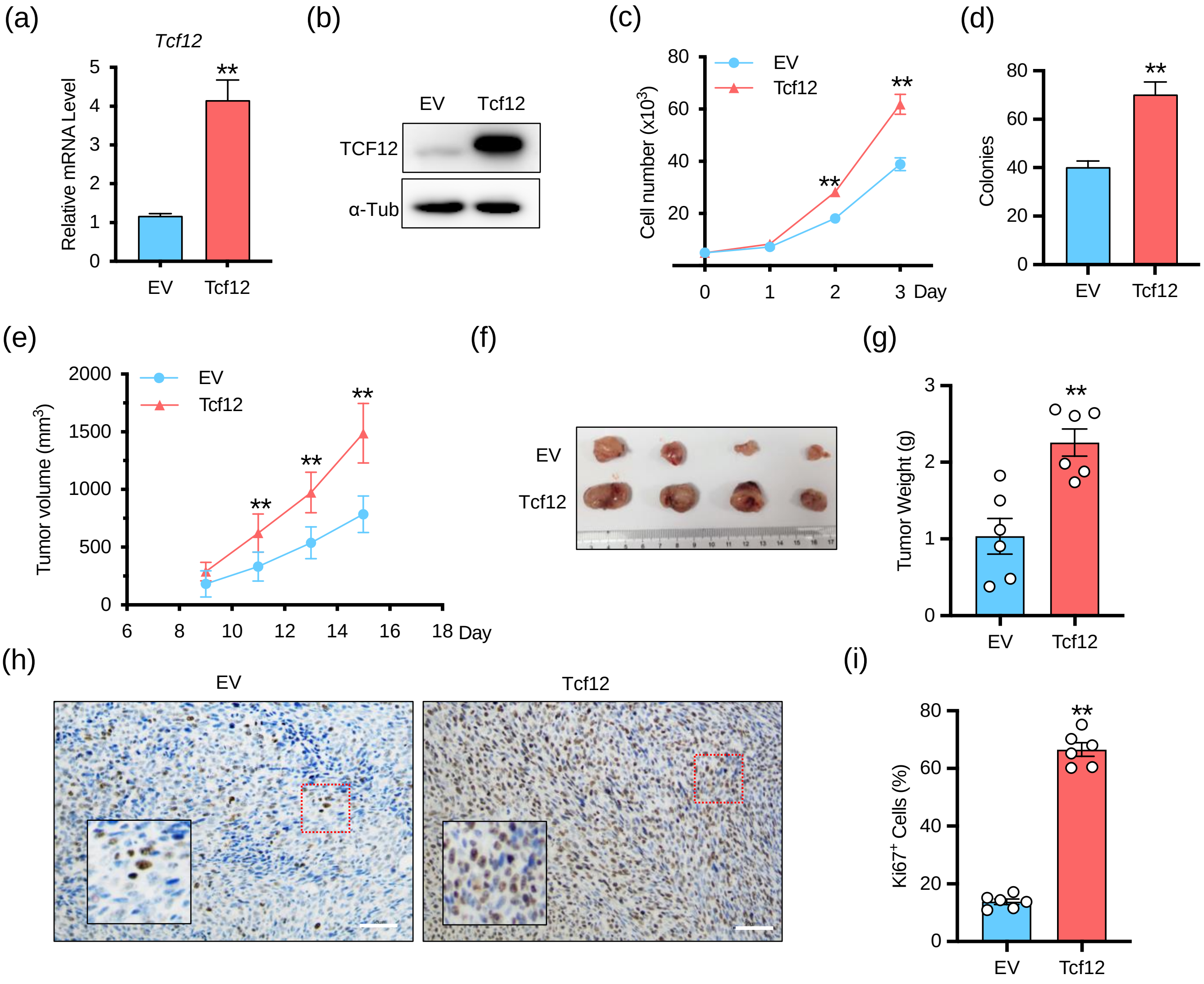

Figure S2. Overexpression of TCF12 enhances melanoma cell proliferation in vitro and tumorigenicity *in vivo*: (**a**,**b**) qPCR (**a**) and immunoblot (**b**) analysis of TCF12 level in YUMM1.7 cell lines following TCF12 overexpression. EV: empty vector expression, Tcf12: mouse TCF12 plasmid overexpression. (**c**,**d**) Analysis of cell proliferation (**c**) and colony formation capability (**d**) in the TCF12 overexpression cells as compared to control cells; (**e**) Tumor growth curves in mice injected with YUMM1.7 cells with TCF12 overexpression; (**f**) Representative tumor image from control (EV) and TCF12 overexpression (Tcf12) mice; (**g**) Tumor weights comparison between control and TCF12 overexpression groups; (**h**,**i**) Ki67 immunohistochemistry staining representative images (red square, 200×; black square, 400×) (**h**) and subsequent analysis (**i**) in tumor tissues. Scale bar: 50 μm. Statistical significance is based on comparison with EV group. * $p < 0.05$, ** $p < 0.01$.
