# Supplementary material for: TCF12 Activates TGFB2 Expression to Promote the Malignant Progression of Melanoma
Source: Cancers (Basel). 2023 Sep 11;15(18):4505. doi: 10.3390/cancers15184505 (PMC10527220; doi:10.3390/cancers15184505)
Supplement: Supplementary file 1 [file cancers-15-04505-s001.zip › Figure S3.pdf]

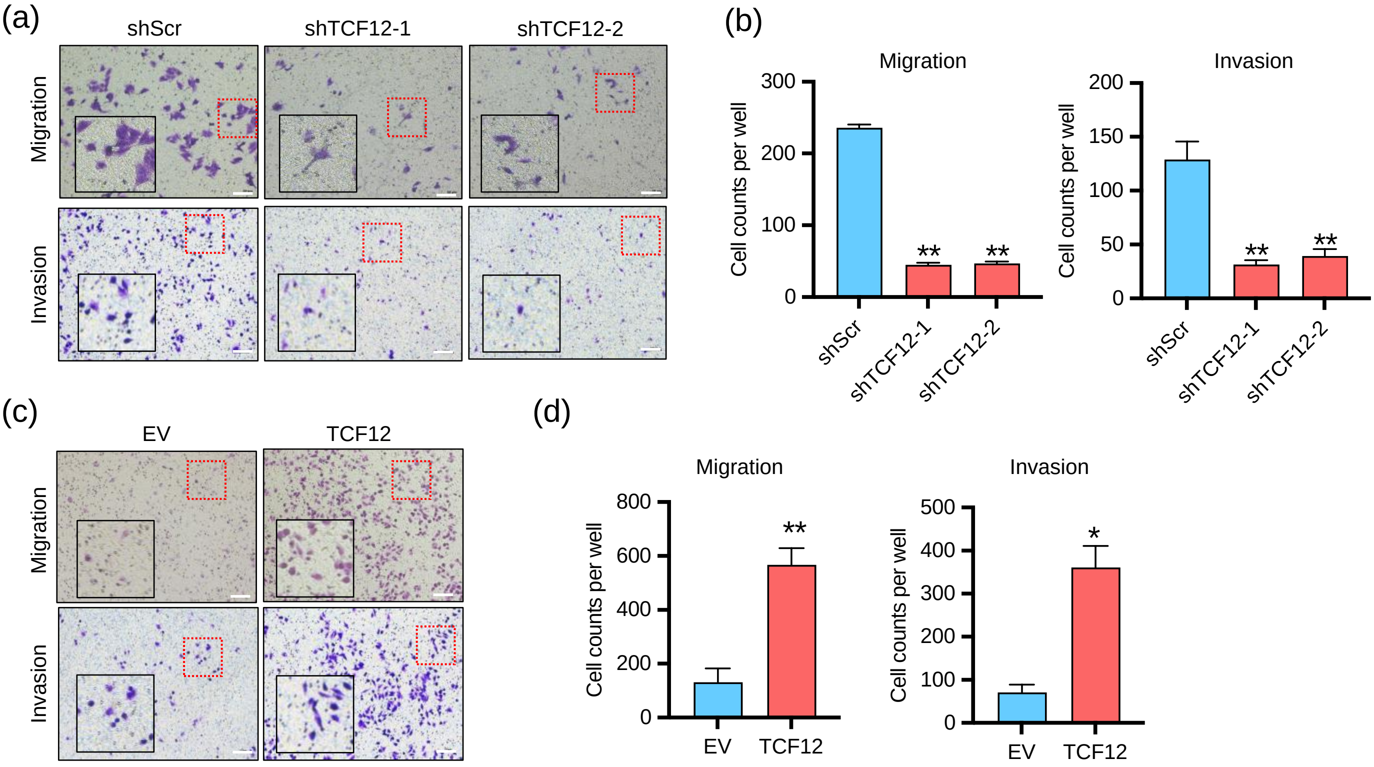

Figure S3. Overexpression of TCF12 promotes melanoma cell migration and invasion *in vitro*: (**a**,**b**) The representative images (red square, 200×; black square, 400×) (**a**) and cell count analysis (**b**) of transwell migration and matrigel invasion assays of A375 cells after TCF12 knockdown. shScr: control shRNA, shTCF12-1/2: human TCF12 specific shRNA; (**c**,**d**) The representative images (red square, 200×; black square, 400×) (**c**) and cell count analysis (**d**) of transwell migration and matrigel invasion assays of A375 cells following TCF12 overexpression. EV: empty vector expression, Tcf12: human TCF12 plasmid overexpression. Statistical significance is based on comparison with shScr group or EV group. * $p < 0.05$, ** $p < 0.01$.
