# Supplementary material for: TCF12 Activates TGFB2 Expression to Promote the Malignant Progression of Melanoma
Source: Cancers (Basel). 2023 Sep 11;15(18):4505. doi: 10.3390/cancers15184505 (PMC10527220; doi:10.3390/cancers15184505)
Supplement: Supplementary file 1 [file cancers-15-04505-s001.zip › Figure S4.pdf]

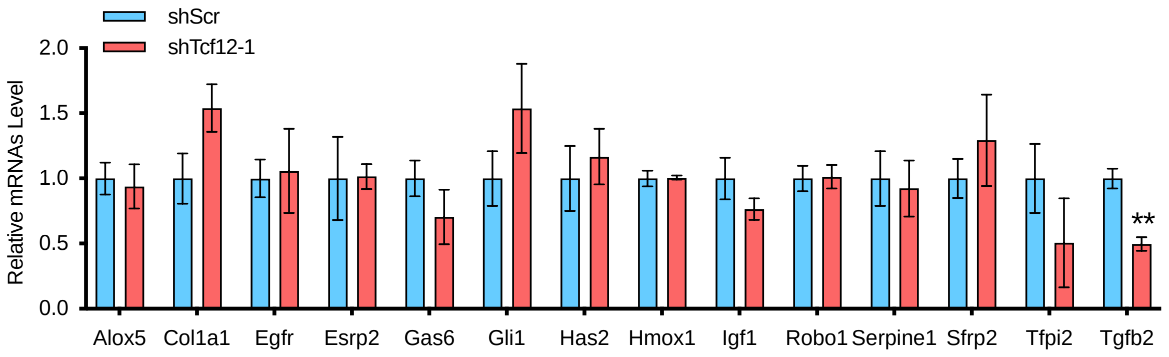

Figure S4. Analysis of potential melanoma-related target genes of TCF12: Expression analysis of a set of melanoma-related target genes with potential TCF12 binding sites in YUMM1.7 cells after TCF12 knockdown. Relative mRNA levels were quantified by RT-qPCR. Statistical significance is based on comparison with control shRNA (shScr) group. ** $p < 0.01$.
