# Supplementary material for: TCF12 Activates TGFB2 Expression to Promote the Malignant Progression of Melanoma
Source: Cancers (Basel). 2023 Sep 11;15(18):4505. doi: 10.3390/cancers15184505 (PMC10527220; doi:10.3390/cancers15184505)
Supplement: Supplementary file 1 [file cancers-15-04505-s001.zip › Figure S5. Original WB images.pdf]

Figure 2b

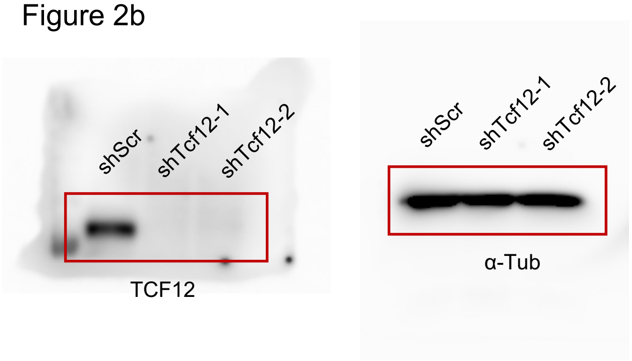

Figure S1b

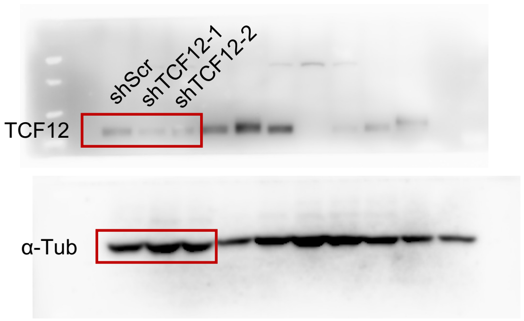

Figure 4e

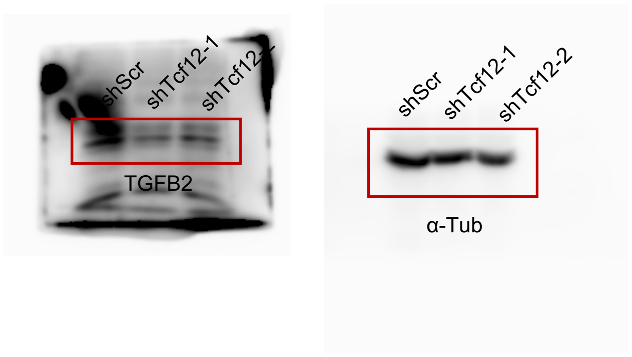

Figure S1f

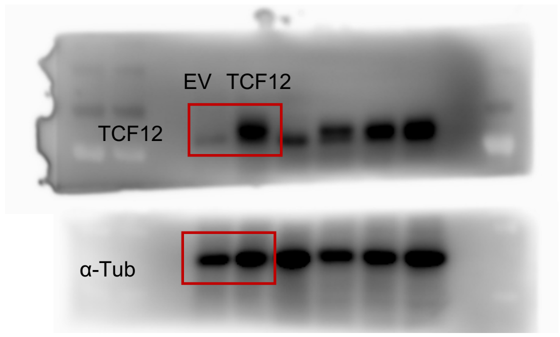

Figure 7b

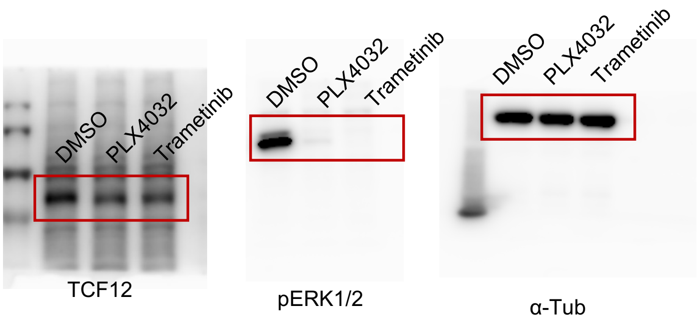

Figure S2b

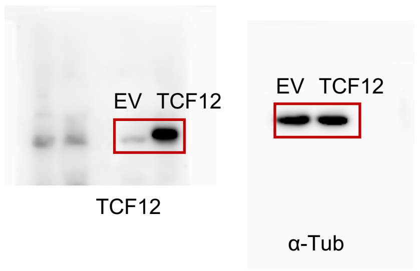

Figure 7c

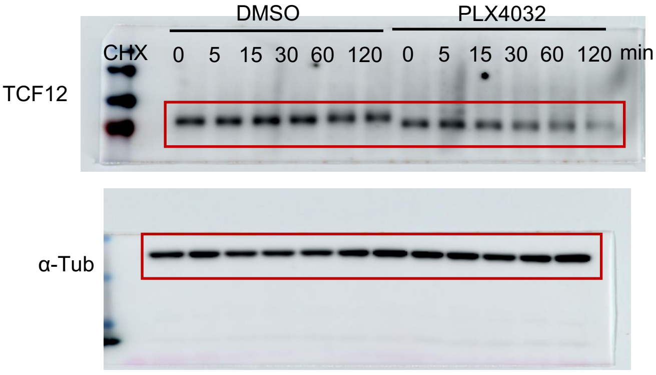

Figure 7e

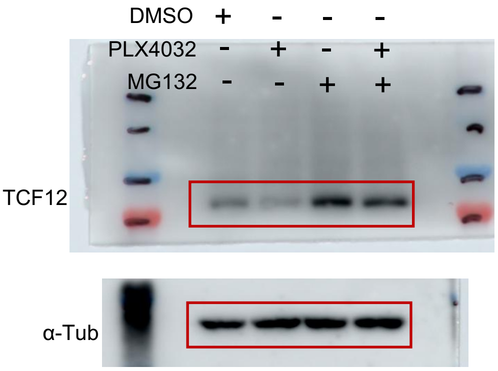

Figure S5. The original western blot figures.
