# Supplementary material for: TCF12 Activates TGFB2 Expression to Promote the Malignant Progression of Melanoma
Source: Cancers (Basel). 2023 Sep 11;15(18):4505. doi: 10.3390/cancers15184505 (PMC10527220; doi:10.3390/cancers15184505)
Supplement: Supplementary file 1 [file cancers-15-04505-s001.zip › Supplementary Tables S1-S3.pdf]

Supplementary tables (Table S1-S3)

Table S1. The primer sequences used for RT-qPCR

Name	Primer Sequence (5'-3')
TCF12	F: GGAAGGACTTGGTTGACCACT
	R: GACCAACTACACTGGGAAGCA
Tcf12	F: CGCGATAGGGACCGACAAG
	R: ACCCGCTGAACTGACTACTTC
Tgfb2	F: TCGACATGGATCAGTTTATGCG
	R: CCCTGGTACTGTTGTAGATGGA
Alox5	F: GGGCTGTAGCGAGAAGCATC
	R: CACGGTGACATCGTAGGAGT
Col1a1	F: GCTCCTCTTAGGGGCCACT
	R: ATTGGGGACCCTTAGGCCAT
Egfr	F: GCCATCTGGGCCAAAGATACC
	R: GTCTTCGCATGAATAGGCCAAT
Esrp2	F: CCAGATCCCGCAGTAGACTC
	R: AGGATTAAGTCGGTCTCGTCC
Gas6	F: CCGCGCCTACCAAGTCTTC
	R: CGGGGTCGTTCTCGAACAC
Gli1	F: CCAAGCCAACTTTATGTCAGGG
	R: AGCCCGCTTCTTTGTTAATTTGA
Has2	F: GTACGGTGCCTTTTTAGCCTC
	R: TAATCGGGGTTTCAAGGGACT
Hmox1	F: AGGTACACATCCAAGCCGAGA
	R: CATCACCAGCTTAAAGCCTTCT
Igf1	F: GTGAGCCAAAGACACACCCA
	R: ACCTCTGATTTTCCGAGTTGC
Robo1	F: CCTTCAGACCTGATCGTCTCC
	R: TGAGCGCGGGTCATCTTTG
Serpine1	F: TCTGGGAAAGGGTTCACTTTACC
	R: GACACGCCATAGGGAGAGAAG
Sfrp2	F: GCCAGCCCGACTTCTCCTA
	R: CCGCATGTTCTGGTACTCGAT
Tfpi2	F: GTGGGCTCCGTTCTTGGTC
	R: AAGCAGCCTCCATAGTTGAATC
36B4	F: GCAGACAACGTGGGCTCCAAGCAGAT
	R: GGTCCTCCTTGGTGAACACGAAGCCC
Gapdh	F: AGGTCGGTGTGAACGGATTTG
	R: GGGGTCGTTGATGGCAACA

Table S2. The primer sequence used for ChIP-qPCR

Name	Primer Sequence (5'-3')
Tgfb2 promoter	F: GGTCCTCCTTGGTGAACACGAAGCCC
	R: GGGTCTTCTGTGTGTTCTGAAATC
Negative region	F: CCTACAGACTGGAGTCACAACA
	R: GCACAGAAGTTAGCATTGTACC

Table S3. Differentially expressed gene after TCF12 knockdown

	No	Symbol	Ensembl Gene ID	Log_2FC	*p*-value
Down-regulated	1	Pcdha9	ENSMUSG00000103770	-7.293	4.74E-05
	2	Tmem30b	ENSMUSG00000034435	-6.447	2.97E-06
	3	Gm14549	ENSMUSG00000084990	-6.290	0.010876
	4	Cox7b2	ENSMUSG00000049387	-6.268	1.29E-05
	5	Pmp2	ENSMUSG00000052468	-6.080	0.015905
	6	Adamts3	ENSMUSG00000043635	-5.941	0.000181
	7	Tnmd	ENSMUSG00000031250	-5.935	0.003778
	8	Krtdap	ENSMUSG00000074199	-5.921	0.022054
	9	Nxf3	ENSMUSG00000057000	-5.724	0.030203
	10	Gm4861	ENSMUSG00000055138	-5.721	0.004613
Up-regulated	1	Lmod2	ENSMUSG00000029683	7.510	0.026541
	2	Lmod3	ENSMUSG00000044086	7.442	0.027945
	3	Trim54	ENSMUSG00000062077	7.230	7.75E-05
	4	Sypl2	ENSMUSG00000027887	7.012	0.038398
	5	Tnnc2	ENSMUSG00000017300	6.831	0.029099
	6	Smtnl1	ENSMUSG00000027077	6.715	0.004863
	7	Adprhl1	ENSMUSG00000031448	6.663	0.005211
	8	Mypn	ENSMUSG00000020067	6.654	0.006609
	9	Kcnt1	ENSMUSG00000058740	6.640	0.005153
	10	Scn3a	ENSMUSG00000057182	6.602	0.005542
